# Diffuse optical microscopy for quantification of depth-dependent epithelial backscattering in the cervix

**DOI:** 10.1117/1.JBO.21.6.066001

**Published:** 2016-06-01

**Authors:** Nico Bodenschatz, Sylvia Lam, Anita Carraro, Jagoda Korbelik, Dianne M. Miller, Jessica N. McAlpine, Marette Lee, Alwin Kienle, Calum MacAulay

**Affiliations:** a Institut für Lasertechnologien in der Medizin und Meßtechnik, Helmholtzstr. 12, D-89081 Ulm, Germany; b British Columbia Cancer Research Centre, Cancer Imaging Department, 675 West 10th Avenue, Vancouver, BC V5Z 1L3, Canada; c University of British Columbia, Division of Gynaecologic Oncology, 2775 Laurel Street, Vancouver, BC V5Z 1M9, Canada

**Keywords:** spatial frequency domain imaging, light scattering, backscattering, structured illumination, subdiffusive scattering

## Abstract

A fiber optic imaging approach is presented using structured illumination for quantification of almost pure epithelial backscattering. We employ multiple spatially modulated projection patterns and camera-based reflectance capture to image depth-dependent epithelial scattering. The potential diagnostic value of our approach is investigated on cervical *ex vivo* tissue specimens. Our study indicates a strong backscattering increase in the upper part of the cervical epithelium caused by dysplastic microstructural changes. Quantization of relative depth-dependent backscattering is confirmed as a potentially useful diagnostic feature for detection of precancerous lesions in cervical squamous epithelium.

## 1 Introduction

Cancerous and precancerous tissues are associated with a number of microstructural changes, which are commonly used for histopathologic diagnosis.^1^ Such alterations in cellular differentiation and subcellular architecture are often microscopically observable long before growth into invasive cancer.^2^ Early detection of cancers or at-risk precancers significantly increases the chance for successful treatment outcome. It is thus highly desirable to visualize pathologic microstructural features *in vivo* both as part of cancer screening or on-site tumor margin assessment.^3^ In addition to its immanent diagnostic benefit, such *in vivo* pathology has the potential to reduce the physical and psychological patient burden by avoiding unnecessary biopsies or potential follow-up surgeries.

The *in vivo* detection of common neoplastic features like changes in both nuclear morphology and chromatin texture, increased DNA content, or lack of cell differentiation is technically very challenging. Nevertheless, current endomicroscopy systems are capable of resolving the morphology of single nuclei and cells with decent image quality.^4^^–^^9^ The rather small imaging field of view of these *in vivo* microscopy devices is, however, suited only for small-area inspection. Furthermore, image interpretation by an experienced clinician during or after image capture is necessary.

An alternative yet more indirect way of measuring tissue microstructure is to study the tissue’s light-scattering properties. Several theoretical and experimental studies suggest that the microscopic and submicroscopic refractive index variations within the cell are the dominant sources of cellular scattering, rather than the shape of the cell and that of its organelles.^10^^–^^12^ While the forward directed part of cellular scattering is related to larger-sized structures, cellular backscattering was found to mostly originate from structures in the range of one quarter to one half of the wavelength.^13^ Owing to the comparably high optical density of the nucleus, cellular light backscattering is mostly influenced by the size of the nucleus and its chromatin texture.^14^^–^^17^ This offers the chance to detect dysplasia or cancer-related morphological changes through quantification of tissue light backscattering.^18^

Several experimental tissue studies report significant cancer-induced changes in the reduced scattering coefficient $\mu_{s}^{\prime }$. However, the direction of change is found to differ with tissue type, as the neoplastic process can also differ between tissue types.^19^^–^^22^ As an example, cancerous versus normal prostate tissue was reported to exhibit an increase in $\mu_{s}^{\prime }$ by ∼25%,^19^ while nonmelanoma skin cancer was observed to produce a decrease in scattering compared to normal skin by ∼25%.^20^

Most cancers originate from epithelium, and, therefore, early detection of cancer by optical means must focus on the optical properties of superficial tissue layers.^23^^–^^33^ Surface sensitivity can be obtained through polarization gated spectroscopy,^23^ or single- and multifiber reflectance spectroscopy^24^^–^^30^ including differential path length spectroscopy.^31^ As a nondiffuse optical imaging modality, optical coherence tomography features high epithelial contrast^33^ and represents another well-suited yet more expensive means for the detection of tissue microstructure.

The above-mentioned diffuse optical imaging modalities are mostly designed as point probes and often have limited control over the actual penetration depth related to their signals. Especially for layered tissue with a mostly complicated tissue structure, this undermines the quantification and analysis of isolated epithelial scattering. In spite of reportedly high sensitivity and specificity values for detection of precancer,^34^^,^^35^ most spectroscopic techniques feature a comparably large volume of optical interrogation and are thus unable to derive meaningful microstructural information. Instead, multivariate data analysis is often used, thus avoiding false assumptions and complex tissue modeling.^28^^,^^32^

In this study, we report a new endoscopic imaging modality for detection of surface scattering, which allows for imaging of almost pure epithelial scattering contrast through the use of structured illumination. Similar to spatial frequency domain imaging (SFDI)^36^ or structured illumination microscopy (SIM),^37^ we employ multiple laterally structured sinusoidal projection patterns to quantify tissue light backscattering. Variation of the projection frequency allows for control over the depth sensitivity, which turns the presented imaging modality into a potentially useful device for cancer screening, margin assessment, or superficial tumor detection through measurement of absolute and depth-dependent backscattering. Quantitation of epithelial backscattering is demonstrated on resected cervical tissue and compared with corresponding histopathologic diagnosis and imaging of the same tissue.^38^

## 2 Experimental Approach

SFDI and SIM both employ projections of laterally structured illumination patterns and thereby achieve imaging depth control. The two approaches operate in different projection frequency regimes. SFDI typically uses frequencies f in the range $0\;{\mathrm{mm}}^{-1}\leq f\leq 0.3\;{\mathrm{mm}}^{-1}$ for derivation of absorption and scattering parameters in turbid media, while SIM employs frequencies above $f\approx 1000\;{\mathrm{mm}}^{-1}$ for optical sectioning and surpassing of the diffraction limited resolution.

Our approach aims at the intermediate frequency regime of about $2\;{\mathrm{mm}}^{-1}\leq f\leq 20\;{\mathrm{mm}}^{-1}$ keeping the effective penetration depth low, while allowing for multiple light scattering to occur. In doing so, we seek to design a diffuse optical imaging method that meets the clinical requirement for maximized epithelial sensitivity for *in vivo* detection of precancerous conditions.

**Fig. 1:**
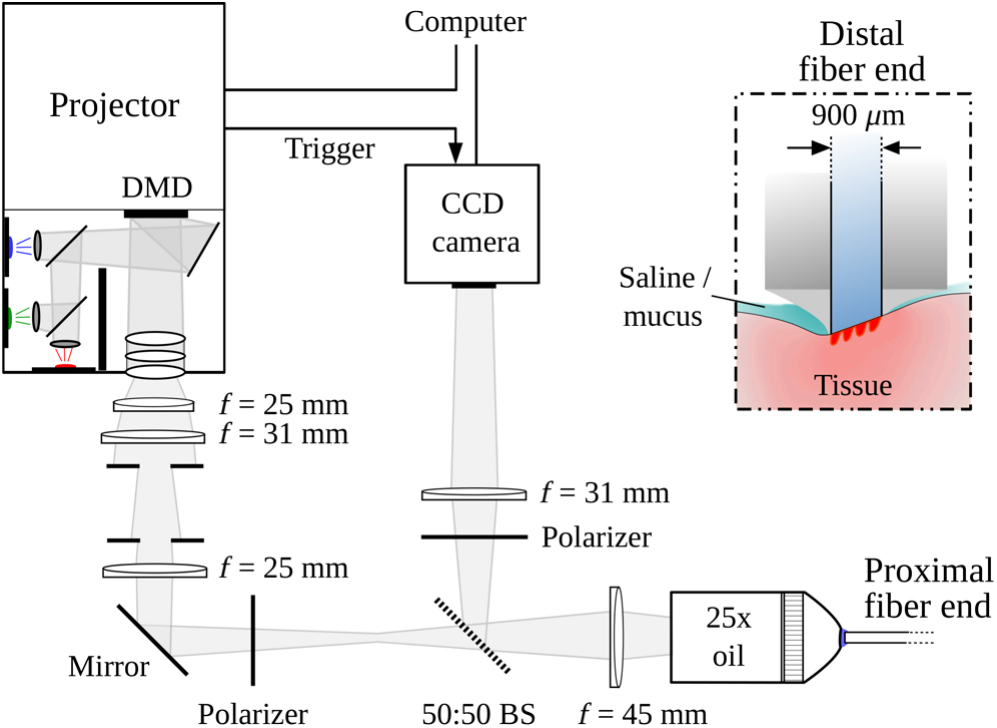
Schematic diagram of the optical setup for projection of structured illumination patterns and simultaneous detection of tissue reflectance. The distal end of the image guide for projection and detection is shown in the inset and is placed in direct tissue contact during measurement.

**Fig. 2:**
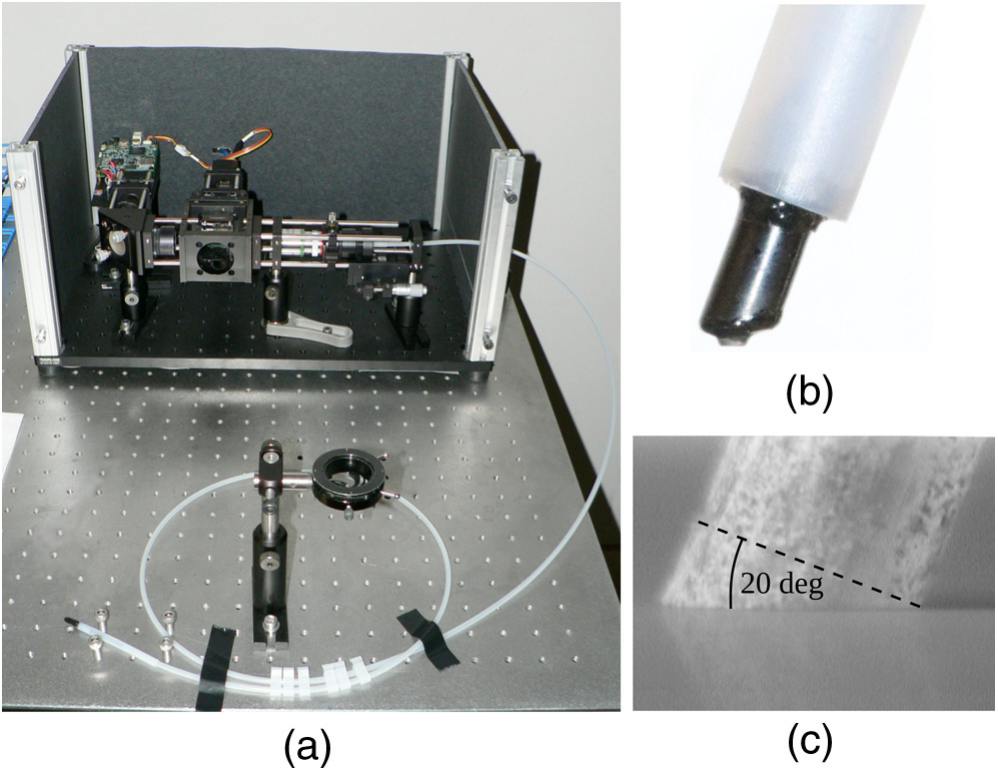
Photograph of (a) our experimental setup and (b) the probe head of the distal fiber end, (c) which is polished at an angle of 20 deg to prevent terminal fiber reflections.

Apart from high optical sensitivity, *in vivo* imaging techniques require ease and flexibility of use and need to address specular surface reflections, which frequently impair quantitative imaging. Our system design is an adaption to these needs and utilizes a flexible image guide for projection and detection of light. Figure 1 provides a schematic diagram of our imaging system, and Fig. 2(a) displays a picture of the system with the employed fiber bundle.

Monochromatic two-dimensional light patterns (256 gray levels) with sinusoidal modulation along one axis and iso-intensity along the other axis are produced by a digital light projector (DLP LightCrafter, Young Optics Inc., Taiwan) at a wavelength of λ=621  nm (FWHM=31  nm). To compensate for the long distance projection design of the device’s light-emitting diode based light engine, a series of four doublet lenses de-magnifies the projection into a 25× oil immersion microscope objective (Zeiss Plan-Neofluar, Germany). The polished proximal end of the image guide (FIGH-30-850N, Myriad Fiber Imaging, Massachusetts), comprising 30,000 imaging fibers with an overall diameter of 900  μm, is placed at the working distance of the objective. Filtered standard microscopy oil is used between objective and fiber bundle to reduce specular reflections at the proximal fiber end and from within the oil. The 1.65-m-long image guide transmits the projection pattern to the tissue. The distal fiber end [see inset of Figs. 1 and 2(b)] is placed in contact with the tissue where saline or epithelial mucus act as index matching fluids. To almost completely suppress specular reflections from the tissue–fiber interface, the distal fiber end is polished at an angle of 20 deg [see Fig. 2(c)].^39^

The incident light pattern interacts with the microscopic tissue structure and is in part diffusely backscattered into the image guide. The fiber projection and detection numerical aperture in air has been measured to be 0.27±0.02 and individual fibers have a near Gaussian shaped angular projection and detection probability. A 50∶50 beam splitter partly directs the backscattering signal to an 8-bit CCD camera (1280×1024, STC-MBE132U3V, Sentech, Japan). The tissue backscattering is weak compared to specular reflections within the objective and at the proximal fiber end. We therefore employ a pair of crossed linear polarizers to considerably reduce these parasitic reflections. The image guide is, however, not polarization maintaining, thus rendering the tissue reflectance image polarization insensitive.

Both projector and CCD camera are computer controlled via USB using a custom-built software, and image capture is projection-synchronized by a trigger connection. This allows for an imaging and projection frame rate of about 15 images per second.

## 3 Image Acquisition and Data Characteristics

The benefit of structured illumination lies in the possibility to subtract the diffuse imaging background caused by deeply penetrating light. Propagation depths of diffuse light in tissue for near-infrared light typically range a few millimeters.^40^ The relative contribution of diffuse background scattering is consequently much higher than the immediate backscattering from the epithelial layer. For our setup, the epithelial contribution to the backscattering signal is roughly about 10% subject to the tissue optical properties and the epithelial thickness. It is also worth noting that larger projection and imaging fields increase the relative influence of the diffuse background.

**Fig. 3:**
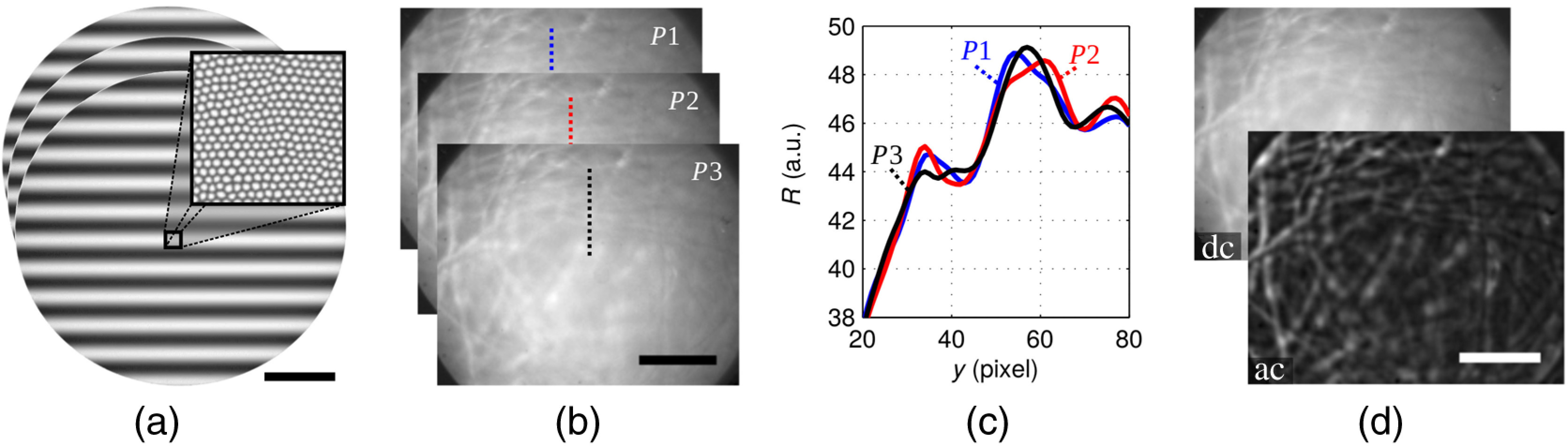
Demonstration of the relative gain in surface sensitivity using structured illumination on moistened lens cleaning paper. (a) Three phase projections at $f=15\;{\mathrm{mm}}^{-1}$, as viewed from the distal end of the fiber bundle. Based on these projection patterns, (b) three phase reflectance images are captured and used to compute (d) a DC background and an AC foreground image. (c) Three line profiles visualize the retained oscillatory signal along the dotted lines of the three reflectance images. Scale bars equal 200  μm.

We demonstrate the subtraction of background scattering with the help of Fig. 3 by imaging multiple layers of moistened lens cleaning paper. Three sinusoidal projection patterns are iteratively projected while shifting the phase of subsequent projections by 120 deg. Figure 3(a) shows three such phase projections at a spatial frequency of $15\;{\mathrm{mm}}^{-1}$ as observed when looking into the distal end of the fiber bundle. The terminal surface of the image guide appears slightly elliptical due to the oblique polishing at 20 deg. We denote the reflectance images corresponding to the three phase projections by P1, P2, and P3. Figure 3(b) illustrates these images for our lens paper experiment. Owing to the preponderance of the diffuse background signal, the illumination pattern is hardly visible in the three reflectance images. Figure 3(c) reveals the residual oscillatory signal through intensity profiles for the three corresponding dotted vertical pixel lines in P1, P2, and P3. The three reflectance images are used to calculate both a DC and an AC image [see Fig. 3(d)] quantifying the average reflectance intensity and the reflectance amplitude at every pixel, respectively. A gain in surface sensitivity is observable in the AC image of Fig. 3(d) by suppression of the diffuse background signal. DC and AC pixel values of both computed images are derived using the following formulas:^36^^,^^37^

$$DC=\frac{1}{3}(P1+P2+P3),$$ (1)

$$\text{AC}=\frac{\sqrt{2}}{3}\sqrt{{\left(P1-P2\right)}^{2}+{\left(P2-P3\right)}^{2}+{\left(P3-P1\right)}^{2}}.$$ (2)

In order to increase the dynamic range of reflectance images, we perform 8×8 software binning on every phase image in a first step. To eliminate residual specular reflections from within the imaging system, we first perform a dark reference for every projection pattern by immersing the probe head into a water-filled black container. The phase intensities captured in this way correspond purely to unwanted system reflections and are subtracted from every subsequent phase image.

The small imaging field of view as constrained by the dimensions of the image guide gives rise to considerable boundary effects.^41^ For the employed spatial frequency regime and especially for low frequencies with only a few oscillations across the image guide, the integral projection intensity of subsequent phase projections differs by a small amount owing to the varying number of projection maxima in the imaging field of view. This can cause considerable oscillatory artifacts in the computed AC image. To circumvent this problem, we perform high-pass filtering^7^ on every phase image before phase demodulation by Eq. (2). The threshold of the employed Butterworth filter of order 2 is set to about one quarter of the projection frequency. This frequency-dependent threshold best minimizes oscillatory artifacts for all projection frequencies while retaining as much low-frequency image information as possible. In spite of high-pass filtering, it is not possible to completely avoid artifacts at the boundaries of the imaging field of view. We therefore restrict our quantitative evaluation to the central imaging field of view in Sec. 5.

To allow for absolute comparison and quantification of reflectance intensities, every tissue measurement is accompanied by a measurement of a freshly diluted emulsion of Intralipid (Fresenius Kabi AG, Germany).^42^ We chose an absolute weight concentration of 7.4% of Intralipid-20% in distilled water to roughly match the observed epithelial backscattering intensity of cervical epithelium. This reference measurement is performed by superficially immersing the probe head into the fat emulsion.^43^

**Fig. 4:**
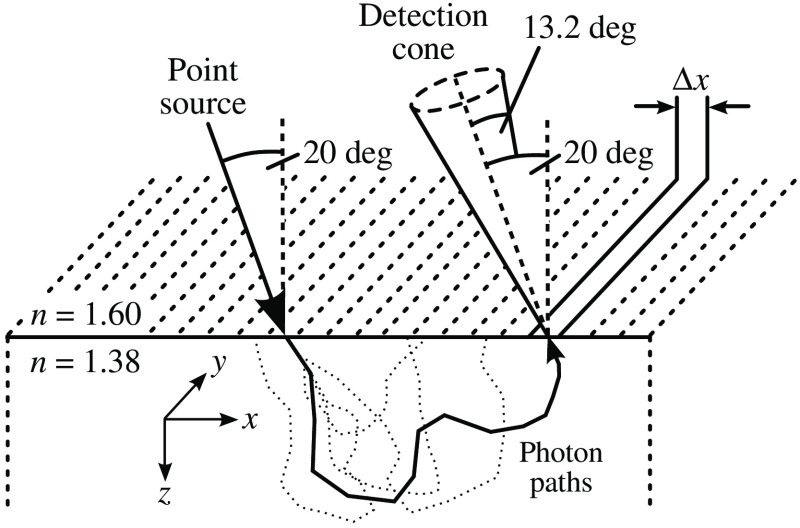
Sketch of the Monte Carlo simulation geometry with oblique point illumination and one-dimensionally binned detection. Photons are detected only if their direction of propagation lies within the angular detection cone upon exiting the semi-infinite medium. This constraint emulates the finite numerical aperture of the imaging fibers.

In order to select spatial frequencies with penetration depths corresponding to that of cervical squamous epithelium, we performed numerous Monte Carlo simulations. Spatial frequency domain reflectance can be most efficiently modeled by Monte Carlo simulations using a point source along with one-dimensionally binned photon detection. We sketch such a simulation geometry in Fig. 4, demonstrating oblique illumination of a semi-infinite scattering medium at an angle of 20 deg. This incidence angle and the upper refractive index of n=1.60^39^ are meant to represent the oblique projection through the image guide. The tissue refractive index is assumed to be n=1.38, and photons are detected within an acceptance angle of 13.2 deg around the projection and imaginary fiber axis, thereby approximating the fiber numerical aperture.

As indicated in Fig. 4, photons are detected in one-dimensional bins, which extend infinitely along the y-axis. The number of detected photons in each bin is normalized to the total number of simulated photons and to the bin width Δx and subsequently referred to as $B_{n}$. We denote the center of each bin with respect to the x-direction as $x_{n}$ with the point of incidence being at x=0. This allows for computation of the spatial frequency domain (SFD) reflectance for any spatial frequency f using the following discrete Fourier transform:

$$R(f)=\overset{N}{\underset{n=0}{\sum }}B_{n}e^{i2\pi fx_{n}}\Delta x.$$ (3)

The number N and spacing Δx of bins was adjusted to cover the entire reflectance area and to allow proper resolution for the frequencies of interest. Note that R(f) in Eq. (3) is a complex number, and to arrive at the AC amplitude value, one has to compute its absolute value.

For Monte Carlo simulations in the studied frequency regime, an optimization that aborts photon migration beyond a certain depth was used to enhance the signal-to-noise ratio of the simulation result. At the same time, we used such an optimization to study the signal penetration depth by monitoring the change in reflectance with different depth constraints.

**Fig. 5:**
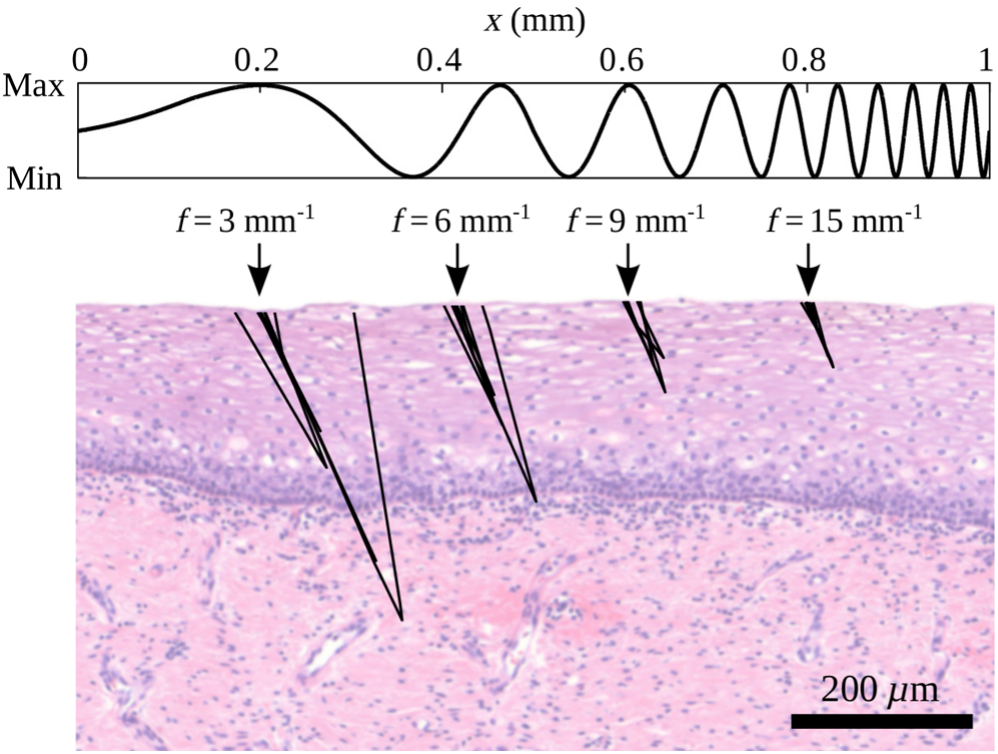
Expected penetration depths illustrated by 10 typical photon paths for each of the four employed spatial frequencies. Modeling is based on Monte Carlo simulations, and the background microscopy image of cervical squamous epithelium puts these penetration depths into experimental context.

Figure 5 depicts 10 typical photon paths for each of the four selected spatial frequencies. These simulations were performed based on phase function data reported and measured by Drezek et al.^11^ for the He-La cervical cancer cell line assuming $\mu_{s}^{\prime }=1\;{\mathrm{mm}}^{-1}$ and $\mu_{a}=0.01\;{\mathrm{mm}}^{-1}$. In order to more easily comprehend the significance of the photon penetration depths, we placed a hematoxylin and eosin (H&E) stained histology image of cervical squamous epithelium with a varying epithelial thickness of ∼200  μm in the background of Fig. 5.

**Fig. 6:**
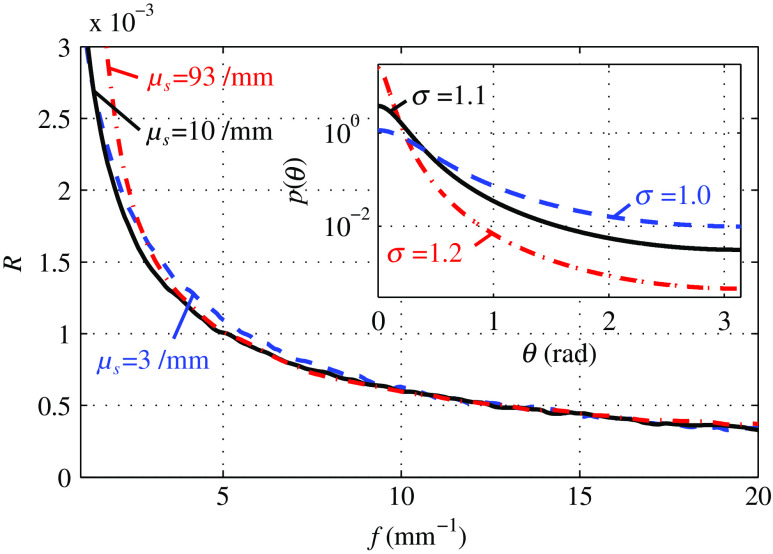
Simulation of SFD reflectance R for the three different scattering phase functions shown in the inset. The scattering coefficient of each simulation is adjusted to obtain matched reflectance at $f=20\;{\mathrm{mm}}^{-1}$. The strong resemblance of curves demonstrates the very similar influence of scattering coefficient $\mu_{s}$ and scattering phase function p(θ).

The penetration depth, especially for the lower spatial frequencies, is observed to depend on the assumed scattering phase function p(θ) and the scattering coefficient $\mu_{s}$. To account for this variability, we state the expected range of the average penetration depth $d_{50}$ and the near maximum penetration depth $d_{95}$ in Table 1. These depth values correspond to the penetration not surpassed by 50 or 95 percent of the photons, respectively. The values were computed by variation of $\mu_{s}^{\prime }$ in the range $1\;{\mathrm{mm}}^{-1}\leq \mu_{s}^{\prime }\leq 2\;{\mathrm{mm}}^{-1}$ and by assuming three different Reynolds-McCormick^44^ scattering phase functions (see inset of Fig. 6 with phase function parameters σ=1.0, 1.1, and 1.2^45^). In this regard, one should be aware of the approximate nature of our simulations, which assume a semi-infinite scattering geometry without depth-dependent variation in scattering.

**Table 1:** Expected ranges of the average $d_{50}$ and near maximum $d_{95}$ penetration depth as derived from numerous Monte Carlo simulations representing assumed optical properties.

f (${\mathrm{mm}}^{-1}$)	$d_{50}$ (μm)	$d_{95}$ (μm)
3	60 to 120	170 to 400
6	40 to 70	130 to 250
9	35- to 50	110 to 150
15	25 to 35	75 to 100

Photon paths in Fig. 5 not only illustrate typical signal penetration depths, but also the scattering angles that are most representative of this light propagation regime.^45^ We find very small and very large scattering angles to dominate the rather straight photon paths. Almost every detected photon undergoes a single high-angle scattering event and a variable number of forward scatterings. The presented photon paths feature low lateral diffusion, which allows for a comparatively high lateral resolution of the reflectance image.^45^

As can be anticipated from the studied photon paths, there are two major influences on epithelial backscattering. The probability for high-angle backscattering as quantified by the scattering phase functions of epithelial scattering particles is one of these major influences. The second major influence is the number of epithelial scattering interactions corresponding to the quantity and cross-section of scattering particles. This second influence is quantified by the scattering coefficient $\mu_{s}$. We find through Monte Carlo simulations that the two influences can hardly be separated by pure study of very high SFD backscattering.

In Fig. 6, we depict the SFD reflectance R, corresponding to the frequency-dependent AC values, for a semi-infinite turbid media with $\mu_{a}=0.01\;{\mathrm{mm}}^{-1}$. Each of the three reflectance curves assumes one of the Reynolds-McCormick scattering phase functions depicted in the inset of Fig. 6. Curves were derived from Monte Carlo simulations according to the previously defined simulation geometry with 100 million simulated photons each. $\mu_{s}$ was adjusted for each simulation, such that the curves coincide at $f=20\;{\mathrm{mm}}^{-1}$. The corresponding $\mu_{s}$ values are depicted next to each curve in the figure. Note that the three curves differ not only in $\mu_{s}$, but also in $\mu_{s}^{\prime }$ with $\mu_{s}^{\prime }(\sigma =1.0)=1\;{\mathrm{mm}}^{-1}$, $\mu_{s}^{\prime }(\sigma =1.1)=1.7\;{\mathrm{mm}}^{-1}$, and $\mu_{s}^{\prime }(\sigma =1.2)=3.7\;{\mathrm{mm}}^{-1}$. Strong similarity of all three curves for most parts of the presented frequency regime exemplifies the difficulty to separate phase function and $\mu_{s}$ influence. This states a major difference to SFDI, where a much lower frequency regime is used for optical parameter separation. Based on the similarity of our imaging approach with both SFDI and SIM, we term this modality diffuse optical microscopy (DOM).

It is also possible to expand the employed frequency regime to lower frequencies for solving the inverse problem. However, unknown layering properties of the epithelium and the underlying stroma accompanied by strong heterogeneity of tissue render this approach infeasible. Nevertheless, we employed this strategy for measurement of polystyrene particle suspensions and for Intralipid emulsions and achieved separation of scattering intensity $\mu_{s}^{\prime }$ and phase function parameter σ,^45^ thereby verifying our setup.

It might occur to the reader that there is the possibility of measuring a phase shift between the projected and reflected sinusoidal light pattern due to the oblique projection in connection with the obliquely polished fiber end. Contrary to intuition, the image guide is almost insensitive to any phase shifts, as the axes of projection and detection coincide and as the detection aperture is small. Therefore, light featuring a single or few scattering interactions can hardly induce phase shifts, as it has to exit the medium in a direction almost parallel to the fiber axes to allow for detection (see photon paths in Fig. 5).

## 4 Tissue Handling and Experiments

We performed *ex vivo* DOM on clinically resected cervical tissue from 18 patients as approved by the British Columbia Cancer Agency under the ethics number H09-03303. These patients were subject to a loop electrosurgical excision procedure (LEEP) following colposcopic examination and a preceding abnormal Pap smear result. Patients were in the age range of 25 to 60 years and had consented to the use of their tissue for biophotonic imaging prior to histopathology processing. The diameter of tissue samples was ∼13  mm with some patient variability. Prior to resection, Lugols iodine and/or acetic acid were applied to the tissue to determine the resection margin.^46^^,^^47^ Time between LEEP and DOM was in the range of 30 to 90 min, and, meanwhile, tissue samples were kept in a 0.9% sodium chloride solution (saline), thus canceling staining or acetowhitening effects. For DOM measurements, samples were repeatedly irrigated with saline to ensure a wetted contact of our imaging probe with the tissue surface.

During a time interval of ∼15  min, close to 40 point measurements were performed on a given tissue sample. For each point measurement, SFD reflectance is quantified at the four spatial frequencies of $f=(2.7,5.5,9.0,14.5)\;{\mathrm{mm}}^{-1}$ at λ=621  nm and with the two additional wavelengths of λ=526  nm and λ=458  nm (with FWHM equal to 31, 47, and 36 nm, respectively). The latter two wavelengths were intended for spectrally resolved evaluation of backscattering. This was, however, undermined by occasional blood absorption from within the mucus or tissue.

Subsequent to imaging and before immersing the sample in formalin, LEEP samples were marked with a line of ink to later facilitate co-registration of histology and DOM.

Histopathologic analysis was performed by a board certified pathologist following tissue sectioning and slicing according to a specified radial sectioning protocol. Based on the pathology report and histologic images, diagnostic maps could be created for each LEEP specimen. However, the lateral localization uncertainty of every point measurement of ∼3  mm and the limited number of radial histology sections hampered diagnostic categorization of every imaging point. Therefore, 80% of all point measurements were discarded and only 20% of points could be grouped into one of six diagnostic categories with high certainty.

The six diagnostic categories are benign columnar (BC) epithelium, benign squamous (BS) epithelium, adenocarcinoma *in situ* (AIS), and cervical intraepithelial neoplasia of different grade (CIN1, CIN2, and CIN 3). AIS relates to the dysplastic or precancerous state of columnar epithelium. In its benign state, columnar epithelium is formed by a single layer of cells. Columnar epithelium mostly lines the endocervix, which is characterized by glandular surface structures and subsurface ducts or cysts. Parts of columnar epithelium are often hidden from the surface due to the undulating tissue structure, and potential lesions may thus be occluded by the overall tissue irregularity.

CIN 1 to 3 are lesions associated with squamous epithelium, which are diagnostically grouped into low-grade squamous intraepithelial lesions (LSIL≜CIN1) and precancerous high-grade squamous intraepithelial lesions (HSIL≜CIN2−3). The main feature of an HSIL is the lack of cellular differentiation, which normally occurs during growth from the basement membrane toward the superficial layer. In HSIL, this lack of differentiation is often accompanied by a reduction in epithelial thickness.^48^ Sample histology images of benign and dysplastic areas for both columnar and squamous epithelium are presented in the bottom part of Fig. 10.

The first five LEEP specimens were used for adjustment of various measurement parameters and are therefore not considered for further evaluation. Of the remaining 13 cases, 102 point measurements could be diagnostically categorized as summarized in Table 2.

**Table 2:** Overview of all point scans from different LEEP cases, which could be successfully categorized into one of the six diagnostic categories. These point scans are used for quantitative evaluation.

Case no.	# BC	# AIS	# BS	# CIN1	# CIN2	# CIN3
1	4	0	5	4	0	0
2	2	5	7	0	0	0
3	0	0	0	0	0	0
4	0	0	9	0	7	3
5	0	0	6	0	0	5
6	0	0	9	0	0	0
7	4	0	3	0	0	0
8	0	0	0	0	0	0
9	3	0	2	0	3	1
10	0	0	3	0	0	3
11	5	0	0	0	0	0
12	0	0	1	1	0	0
13	1	0	2	0	0	4
**Sum**	**19**	**5**	**47**	**5**	**10**	**16**

## 5 Results

The main focus of our study is the analysis of squamous epithelium. For completion, most evaluation steps are, however, presented for all six diagnostic categories.

Following the predictions of light propagation models, the nucleus is expected to be the dominant source of backscattering. We expect enhanced backscattering at high spatial frequencies in HSIL owing to the presence of more and enlarged nuclei in its superficial epithelium. In this respect, we seek to confirm the observation of depth-dependent epithelial backscattering as reported in 2012 by Adegun et al.^33^ using optical coherence tomography.

Based on the residual categorization uncertainty of each imaging point listed in Table 2 along with high structural tissue-variability even within the dimensions of the imaging field of view, we perform a statistical evaluation to observe the major precancer-related backscattering effects. Our numerical evaluation averages SFD reflectance over the central circular 50% of the imaging field of view for each point scan, thus neglecting heterogeneity within each point measurement. The narrowed evaluation circle was selected to achieve a higher evaluation accuracy in the absence of residual projection boundary effects. Due to low image quality caused by a low signal-to-noise ratio, we refrained from evaluating image texture features, which might carry additional diagnostic information.

**Fig. 7:**
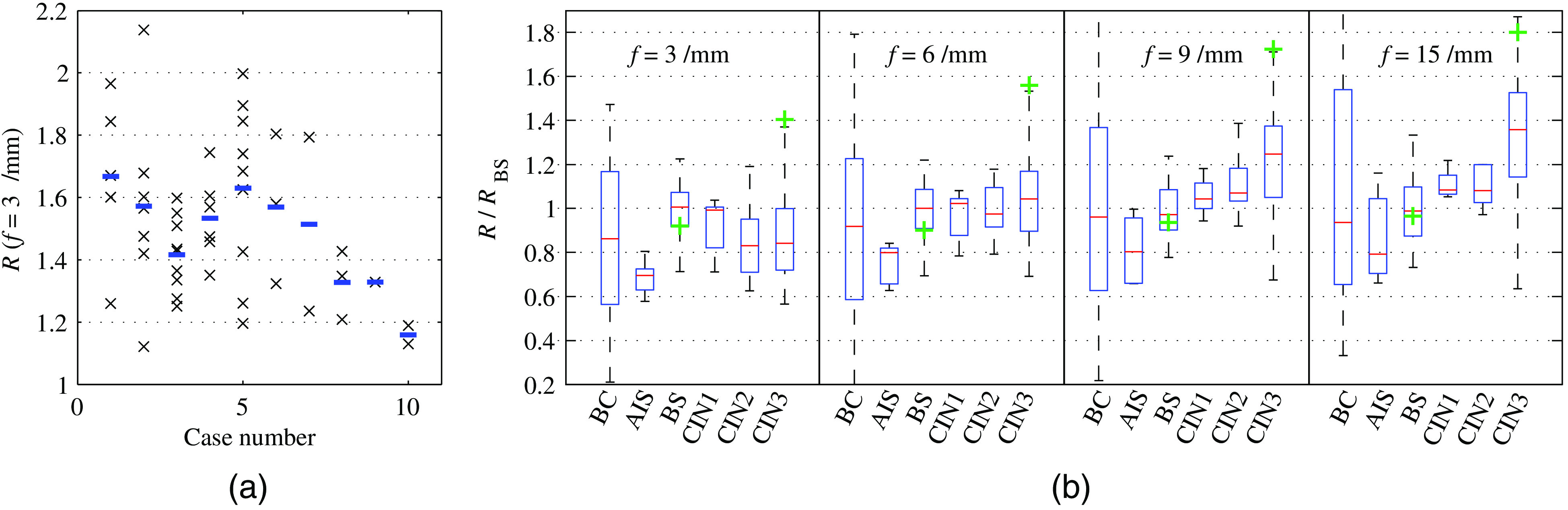
(a) Inter- and intrapatient variability of BS reflectance at $f=3\;{\mathrm{mm}}^{-1}$. (b) The diagnostic group dependent reflectance values for all cases are presented using a boxplot. All point scans within each category are first referenced by their case dependent mean BS reflectance [see blue bars in (a)]. Green crosses indicate the location of median values 1 to 2 min after application of acetic acid.

In a first step, we study the inter- and intrapatient reflectance variability for BS epithelium at $f=3\;{\mathrm{mm}}^{-1}$. We choose this frequency as it is expected to best average over the epithelial thickness (see Fig. 5). Figure 7(a) compares all BS point measurements of the corresponding LEEP specimens. Every black cross corresponds to the mean reflectance value of a single point scan as averaged over the central circular imaging subsection of diameter 600  μm, and blue bars mark the average BS reflectance for each case. The presented intensities refer to the factor by which the tissue reflectance exceeds that of our Intralipid reference with $\mu_{s}^{\prime }=2.0\pm 0.1\;{\mathrm{mm}}^{-1}$.

Figure 7(a) demonstrates a surprisingly large variability in epithelial backscattering both between patients and especially within patients. It is presumably this strong intrapatient variability that decreases the specificity of diagnostic inspection through acetowhitening.^49^ No correlation of BS backscattering could be found with patient age or with distance from the cervical os.

In Fig. 7(b), we depict the reflectance for each diagnostic group and for all four spatial frequencies using a boxplot. In order to reduce the variability induced data spreading, every point measurement is first divided by its corresponding case-averaged reflectance for BS at $f=3\;{\mathrm{mm}}^{-1}$. This explains why the median values (red lines in boxplot) are always found close to one. Boxes in Fig. 7(b) indicate the location of the first and third quartile and whiskers describe the maximum and minimum values of each diagnostic ensemble.

In spite of large overall variability, we observe a slight decrease in reflectance from squamous epithelium at $f=3\;{\mathrm{mm}}^{-1}$ when comparing BS to CIN2 and CIN3 in Fig. 7(b). The opposite trend is found for $f=9\;{\mathrm{mm}}^{-1}$ and $f=15\;{\mathrm{mm}}^{-1}$, where reflectance intensities, on average, increase with the progression from BS toward high-grade dysplasia. The decrease in low-frequency reflectance for CIN2 and CIN3 may be explained by epithelial thinning and a comparably weak backscattering of underlying stroma in spite of the presence of stromal collagen.^11^ The increase in high-frequency reflectance for HSIL presents the expected effect due to low cellular differentiation and lower cytoplasmic-to-nuclear ratios in superficial epithelium. This effect can be increased through application of acetic acid onto the epithelium.^47^

Acetic acid is believed to modify cellular protein and chromatin texture, thereby amplifying refractive index oscillations and backscattering. We investigated this acetowhitening on several of the tissue specimens and reflectance point scans were captured 1 to 2 min after application. Four BS and ten CIN3 point scans could be categorized and quantified for their increase in backscattering. The median reflectance values for these two diagnostic groups during acetowhitening are marked as green crosses in Fig. 7(b). The quantified acetowhitening confirms the reported high sensitivity toward squamous intraepithelial lesions with an average amplification in backscattering by >70% for spatial frequencies of 9 and $15\;{\mathrm{mm}}^{-1}$.

**Fig. 8:**
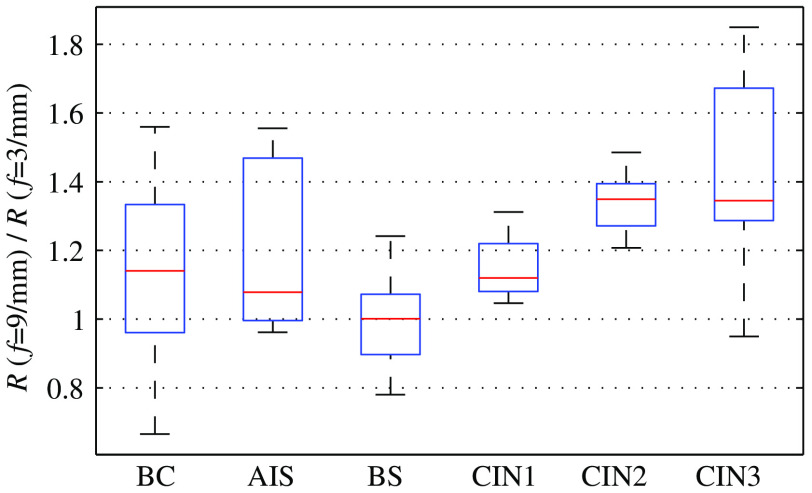
Boxplot for the ratio of SFD reflectance at two spatial frequencies quantifying the depth-dependent variation in epithelial backscattering. The statistics of every diagnostic category represent all corresponding point scans given in Table 2. We find that the backscattering ratio may allow for diagnostic differentiation of squamous epithelium.

**Fig. 9:**
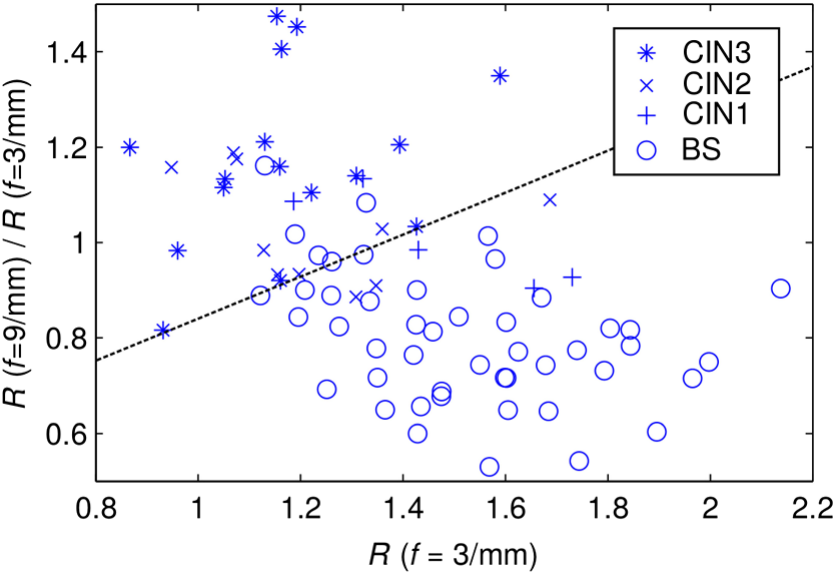
Absolute SFD reflectance ratio for each categorized point scan on squamous epithelium versus low-frequency reflectance. Opposite to the data in Fig. 8, point scans are not referenced to BS epithelium in this plot. A dashed line roughly separates HSIL from LSIL/BS.

The boxplot in Fig. 7(b) confirms the reported malignancy-related variation in depth-dependent epithelial backscattering.^33^^,^^50^ In consequence, we study the ratio of epithelial backscattering at $f=3\;{\mathrm{mm}}^{-1}$ over that at $f=9\;{\mathrm{mm}}^{-1}$ as a potential diagnostic quantifier. This ratio has been computed for every imaging pixel before averaging over points scans and is statistically evaluated in Fig. 8. Prior to evaluation of each diagnostic category, all data have been averaged by its corresponding case-average BS reflectance ratio. Figure 8 demonstrates that the backscattering ratio $R(f=9\;{\mathrm{mm}}^{-1})/R(f=3\;{\mathrm{mm}}^{-1})$ correlates with the different states of squamous epithelium. Characteristic for the backscattering ratio is a decreased sensitivity to both inter- and intrapatient variability as well as to drifts in the light source intensity. In Fig. 9, we plot the ratio intensity for every categorized squamous point scan against reflectance at $f=3\;{\mathrm{mm}}^{-1}$. In this case, data are not referenced against BS epithelium and feature the additional variability between patients.

From Fig. 9, the absolute separation of designated dysplastic point scans becomes apparent. An arbitrary dashed line was drawn in this graph as the basis for a basic sensitivity and specificity estimate pertaining to the detection and differentiation of HSIL from LSIL/BS tissue. We thus arrive at a sensitivity of 88% and a specificity of 87%. Assessment of these numbers should consider the potential heterogeneity in each imaging field of view and the remaining categorization uncertainty for each point scan. It is worth mentioning that all five BS points and one out of the two CIN1 data points above the dashed line (false positives) originate from LEEP samples, which were clinically diagnosed to contain an HSIL.

**Fig. 10:**
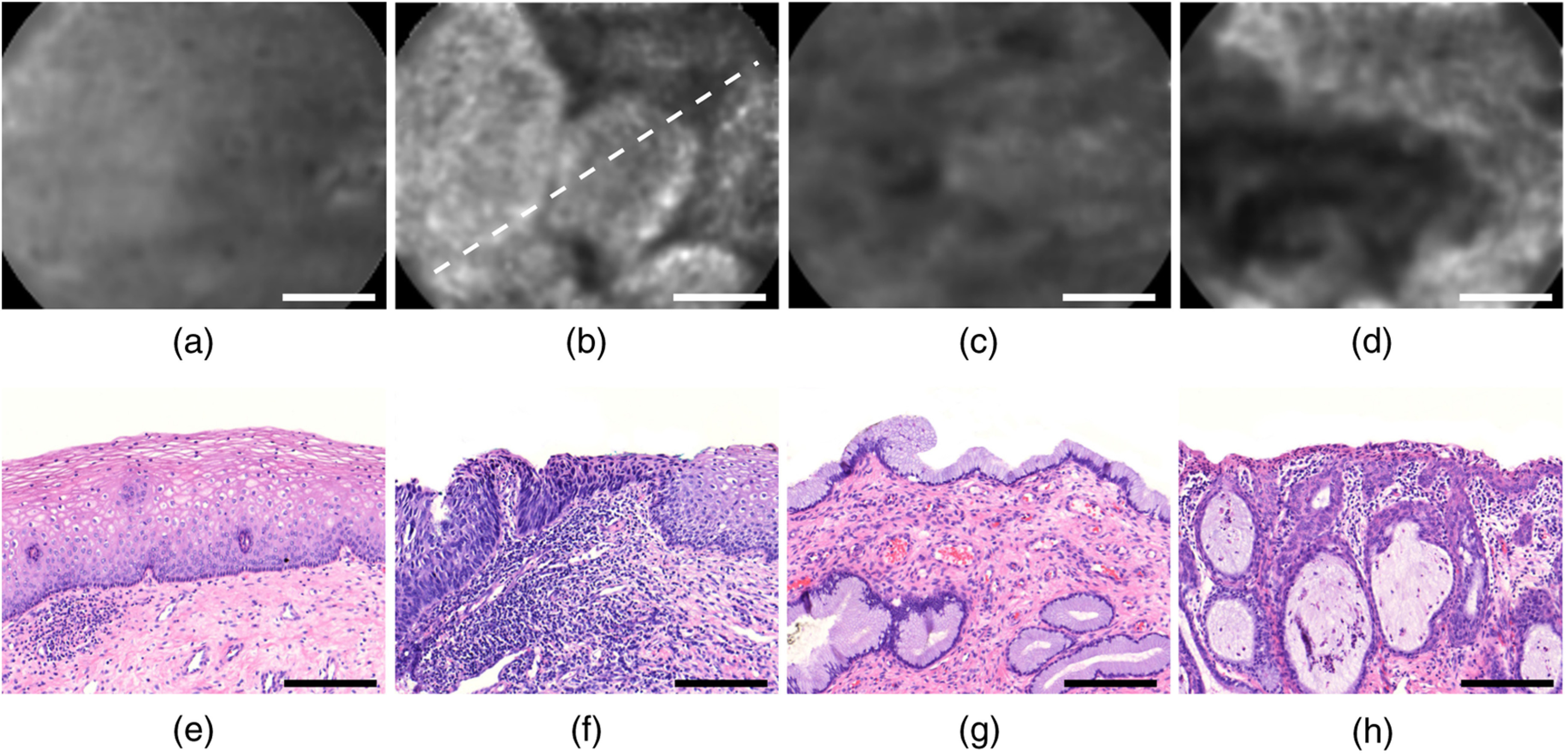
Qualitative comparison of representative DOM images for different diagnostic categories [(a) through (d)] along with corresponding histology sections from a nearby H&E stained tissue slice [(e) through (h)]. DOM images were obtained at $f=9\;{\mathrm{mm}}^{-1}$ and the four image pairs depicted from left to right are BS, CIN3, BC, and AIS. Histology section (f) was successfully matched with the CIN3 image (b), with the dashed line showing the expected direction of sectioning. Scale bars measure 200  μm.

Figures 10(a)–10(d) exemplify reflectance images at $f=9\;{\mathrm{mm}}^{-1}$ for BS, CIN3, BC, and AIS epithelium, respectively. Figures 10(e)–10(h) illustrate nearby histology sections of the same specimen. In spite of some qualitative variability in each diagnostic group, we find lateral homogeneity in backscattering to be a characteristic feature of BS point scans [see Fig. 10(a)]. BC images almost always appear more heterogeneous, presumably owing to the glandular and undulating tissue structure. Often, they are also found to have a lower overall intensity, as can be observed by comparison of Figs. 10(a)–10(d), which feature equal gray scaling. We expect low scattering of stromal tissue to account for lower maximum intensities in BC reflectance images.

Similar image heterogeneity as in BC epithelium with higher maximum reflectance values is observed for AIS [Fig. 10(d)]. Presumably, the higher peak intensities are related to the quasi-stratified columnar cells with enlarged nuclei.

Figures 10(b) and 10(f) relate to CIN3 epithelium and have been successfully co-registered by inking of the tissue scan location, as the ink could later be relocated in the histologic slice. The dashed line in Fig. 10(b) corresponds to the expected direction of histologic sectioning presented in Fig. 10(f) with BS epithelium at the upper right end of the line. In this image, the reflectance $R(f=9\;{\mathrm{mm}}^{-1})$ doubles from the BS to the CIN3 area along the dashed line. We find intensity variations similar to that in Fig. 10(b) also in other HSIL images, which may be explicable by small-scale variations in the extent of dysplastic tissue alterations or by intermittent benign areas. We therefore expect that absolute scattering differences between benign and high-grade dysplastic squamous epithelium are possibly larger than quantified in our statistical approach, which is based on averaging over local heterogeneities.

## 6 Conclusions

Detection and visualization of precancers and preinvasive cancers in cervical squamous epithelium were demonstrated by the use of a fiber optic imaging approach with structural contrast.^6^^,^^7^ Contrary to common endomicroscopic modalities, which aim at maximized resolution for *in vivo* pathology,^3^ we make use of microscopic information encoded in backscattering signals to visualize dysplasia. This allows for potentially much larger fields of views. It was shown that diagnostically valuable and almost pure epithelial and subepithelial backscattering may be quantified using structured illumination. We found high-grade dysplasia to correlate with increased backscattering from within the upper epithelium, thereby confirming theoretical predictions.^14^^,^^16^^,^^22^ This pilot study suggests that the depth dependence of epithelial backscattering may be a meaningful diagnostic property for differentiation of cervical squamous epithelium, which can overcome the observed large inter- and intrapatient variability in backscattering. Our experimental approach may be similarly applicable and potentially useful for squamous epithelium of different origin such as lung, colon, or the oral cavity.

Low overall costs of our optical setup (in the range of 5000 U.S. dollars) may qualify the imaging modality also for developing countries with the highest incidence of cervical cancer and high associated mortality.^49^ By use of diagnostic intensity thresholds, DOM imaging is potentially very easy to interpret and signals may be amplified by the use of acetowhitening. Given its imaging speed and a potentially much larger field of view, DOM might be well suited for large-area tissue screening or for the assessment of resection or tumor margins.

The heterogeneity of cervical epithelium including metaplastic and glandular tissue structures states a major difficulty for the interpretation of imaging data. Larger imaging fields of views enabled by a wider image guide or a potential noncontact colposcopic or endoscopic implementation may help with the overall differentiability. DOM allows for fast and near-real-time imaging and our setup achieved an AC image frame rate of five images per second. However, lateral scanning during capture of successive phase images produces considerable artifacts in derived AC images. Faster imaging and fewer required phase projections may help in reducing these artifacts, thus allowing for rapid inspection of large tissue areas. Further image quality improvement is possible by enhanced suppression of internal specular reflections within the optical setup by using a microscope objective designed for epi-illumination and a camera with higher dynamic range.

We hope that our imaging modality will find use in further tissue studies, potentially confirming the sensitivity to high nuclear density in cancer and in other epithelial dysplasia.
